# SLC22A11 Inserts the Uremic Toxins Indoxyl Sulfate and P-Cresol Sulfate into the Plasma Membrane

**DOI:** 10.3390/ijms242015187

**Published:** 2023-10-14

**Authors:** Maurice Tust, Julian Peter Müller, Dietmar Fischer, Dirk Gründemann

**Affiliations:** Department of Pharmacology, Faculty of Medicine and University Hospital Cologne, University of Cologne, Gleueler Straße 24, 50931 Cologne, Germanyjumueller@ukaachen.de (J.P.M.); dietmar.fischer@uni-koeln.de (D.F.)

**Keywords:** SLC22A11, OAT1, p-cresol sulfate, indoxyl sulfate, LC-MS, chronic kidney disease, membrane insertion, transport mechanism

## Abstract

Chronic kidney disease (CKD) is a global health concern affecting millions worldwide. One of the critical challenges in CKD is the accumulation of uremic toxins such as p-cresol sulfate (pCS) and indoxyl sulfate (IS), which contribute to systemic damage and CKD progression. Understanding the transport mechanisms of these prominent toxins is essential for developing effective treatments. Here, we investigated whether pCS and IS are routed to the plasma membrane or to the cytosol by two key transporters, SLC22A11 and OAT1. To distinguish between cytosolic transport and plasma membrane insertion, we used a hyperosmolarity assay in which the accumulation of substrates into HEK-293 cells in isotonic and hypertonic buffers was measured in parallel using LC-MS/MS. Judging from the efficiency of transport (TE), pCS is a relevant substrate of SLC22A11 at 7.8 ± 1.4 µL min^−1^ mg protein^−1^ but not as good as estrone-3-sulfate; OAT1 translocates pCS less efficiently. The TE of SLC22A11 for IS was similar to pCS. For OAT1, however, IS is an excellent substrate. With OAT1 and p-aminohippuric acid, our study revealed an influence of transporter abundance on the outcomes of the hyperosmolarity assay; very high transport activity confounded results. SLC22A11 was found to insert both pCS and IS into the plasma membrane, whereas OAT1 conveys these toxins to the cytosol. These disparate transport mechanisms bear profound ramifications for toxicity. Membrane insertion might promote membrane damage and microvesicle release. Our results underscore the imperative for detailed structural inquiries into the translocation of small molecules.

## 1. Introduction

**Figure 1:**
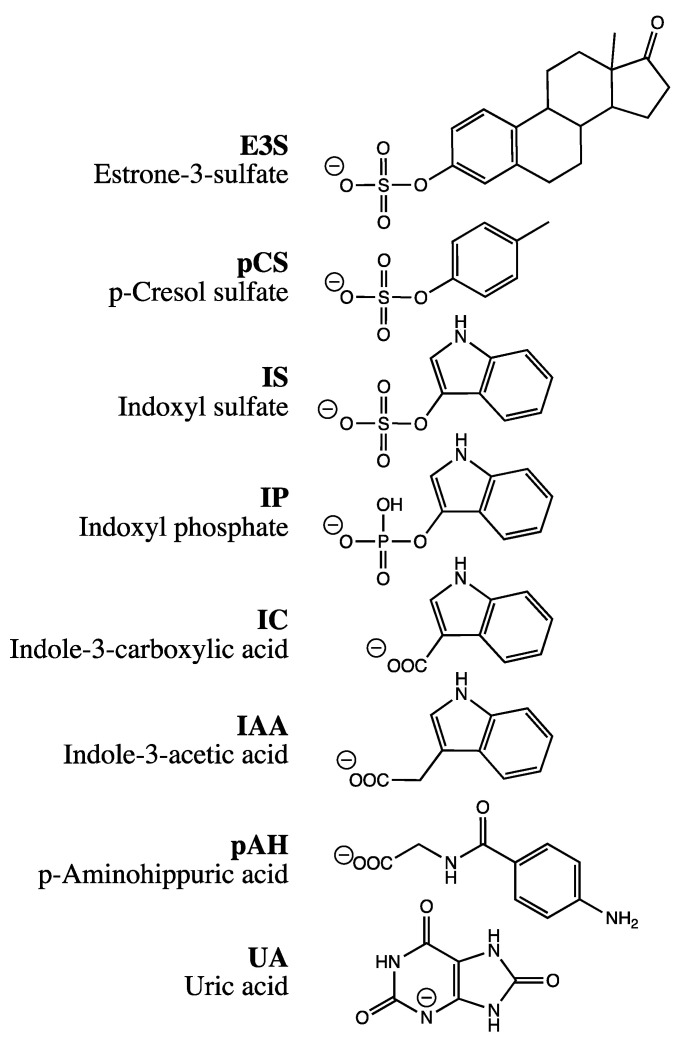
Structures of compounds used for investigation. Charges are drawn to indicate the dominating species at pH = 7.4.

With diminished kidney function, metabolic waste products that are excreted by the kidney under normal conditions accumulate in the body and cause systemic damage. The increased retention of so-called uremic toxins further deteriorates renal function, contributing to the development of chronic kidney disease (CKD) [1,2]. CKD is estimated to affect 8–16% of the population worldwide [3,4]. Largely protein-bound uremic toxins such as p-cresol sulfate (pCS) and indoxyl sulfate (IS) are a major challenge in the treatment of CKD as they are difficult to remove via dialysis [5] and are associated with various co-morbidities including cardiovascular disease and osteoporosis [6]. pCS and IS (see Figure 1 for structures) are formed in the human body by the metabolism of amino acids by intestinal bacteria. Tyrosine and phenylalanine, for example, are degraded to p-cresol [7], which, after absorption, is sulfated in the liver by sulfotransferase 1A1 (SULT1A1) to pCS [8]. In contrast, tryptophan is metabolized by bacterial tryptophanase to indole [9], which is hydroxylated in the liver by cytochrome P450 2E1 (CYP2E1) to 3-hydroxy indole [10] and subsequently sulfated by SULT1A1 to IS [11].

pCS and IS are normally excreted from the human body through the renal proximal tubule. The two toxins are mainly cleared from the blood compartment via uptake catalyzed by organic anion transporter 1 (OAT1) and OAT3 [12,13,14]. OAT1 (human gene symbol *SLC22A6*) and OAT3 (*SLC22A8*) are found in the basolateral membrane of proximal tubular cells; their substrate spectrum, besides pCS and IS, encompasses many other anionic compounds, including metabolites, toxins, and drugs [15]. In addition, IS is a substrate of SLC22A11, also known as OAT4 [13]. SLC22A11 is located in the apical membrane of renal proximal tubule cells and transports uric acid (UA), glutamate, steroid sulfate conjugates such as estrone-3 sulfate (E3S), and other compounds, including drugs and toxins [15,16,17]. Therefore, SLC22A11 was suggested to mediate efflux and reabsorption of IS on the apical side of the proximal tubule [13]. It is unknown whether SLC22A11 transports pCS.

We have previously uncovered that SLC22A11 transports the substrates E3S and UA via fundamentally different mechanisms [18]. Our data suggest that SLC22A11 inserts E3S into the plasma membrane while UA, a regular substrate, is transported into the cytosol [18]. Indeed, some transporters, like the long-chain fatty acid transporter FadL of *Escherichia coli*, can insert certain amphipathic or predominantly hydrophobic molecules into the membrane through a lateral opening [19]. In addition, the ability of ABC transporters to retrieve and transport membrane-soluble substrates from the phospholipid bilayer is well known [20]. Surprisingly, the insertion into the plasma membrane of small molecules by SLC transport proteins has hardly been explored, yet this alternative route has important implications for transcellular trafficking and the intracellular processing of drugs and toxins.

To better understand the pathophysiological mechanisms of toxicity in humans caused by the uremic toxins pCS and IS and their roles in CKD progression, it is important to identify the underlying transport mechanisms in detail. The current belief invariably holds that uremic toxins such as pCS and IS are transported by organic anion transporters into and out of the cytosol. However, our recent results might challenge this assumption: due to the structural similarity to E3S (see Figure 1), transport proteins could insert pCS (in fact, an exact substructure of E3S) and IS into the plasma membrane.

We have recently established a hyperosmolarity assay to distinguish between regular transport of small molecules into the cytosol and insertion into the plasma membrane [21]. In this assay, the uptake of substrates into HEK-293 cells (293 cells) by specific transport proteins is measured in isotonic and hypertonic buffers in parallel. Inhibition of substrate accumulation due to hyperosmolarity indicates transport into the cytosol, whereas stimulation of accumulation indicates insertion into the plasma membrane [21]. The decrease in the accumulation of regular substrates in hyperosmolar buffer might be caused by the reduction in cell volume and endocytosis of plasma membrane areas, including transporters, during membrane remodeling [22,23,24,25,26]. In contrast, the increase in substrate accumulation could be explained by the formation of invaginations and a decrease in membrane tension due to increased extracellular osmotic pressure. These changes in membrane properties could provide more space for small molecules to be inserted into the plasma membrane [21,27].

The primary aim of the present study was to investigate the mechanisms of transport of pCS and IS via human OAT1 and SLC22A11 in 293 cells using the hyperosmolarity assay. Our results indicate that OAT1 transports pCS and IS into the cytosol, whereas SLC22A11 transports pCS and IS into the plasma membrane.

## 2. Results

The mechanisms of transport of the uremic toxins IS and pCS by SLC22A11 and OAT1 were investigated through hyperosmolarity experiments developed previously [21]. Transport proteins were inducibly expressed in 293 cells, which were then incubated in the hyperosmolarity assay; the resulting cell lysates were analyzed via LC-MS/MS. A stimulation of substrate accumulation in the hyperosmolarity assay indicates integration into the plasma membrane, whereas inhibition of substrate accumulation by hyperosmolarity indicates transport into the cytosol [21].

### 2.1. OAT1 Abundance Affects the Hyperosmolarity Assay

**Figure 2:**
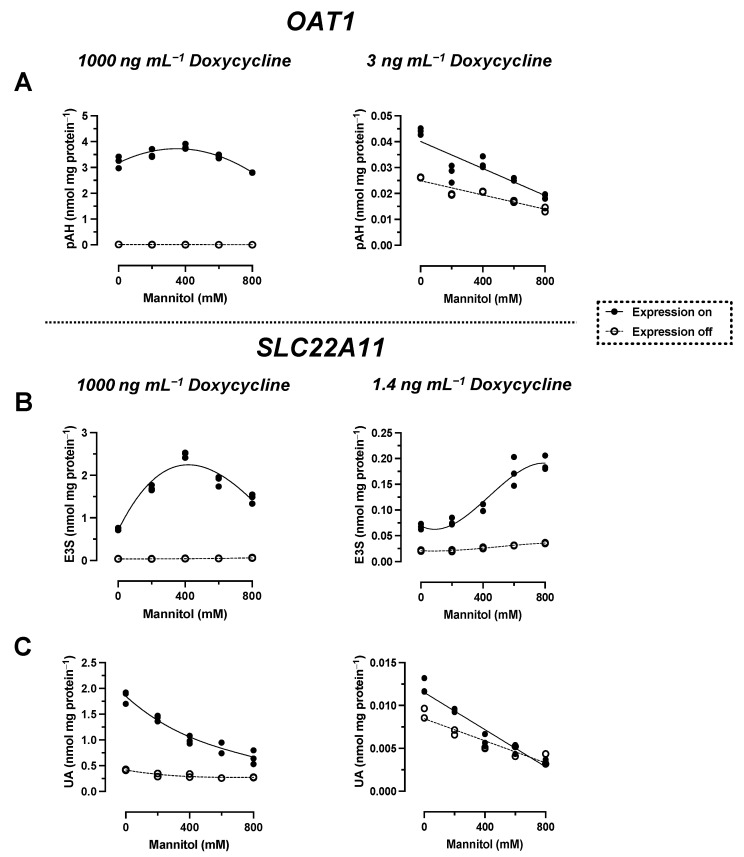
Hyperosmolarity assay with reduced concentrations of doxycycline. The analyte content of cell lysates was determined via LC-MS. Each symbol represents a dish. (**A**) Stably transfected 293 cells with (on; *n* = 3) or without (off; *n* = 2) expression of OAT1 were incubated with 10 µM pAH for 20 min at 37 °C in uptake buffer with mannitol, as indicated. The expression of OAT1 was either normal (1000 ng mL^−1^ doxycycline) or reduced (3 ng mL^−1^ doxycycline). (**B**) Stably transfected 293 cells with (on; *n* = 3) or without (off; *n* = 2) expression of SLC22A11 were incubated with 10 µM E3S for 60 min at 37 °C in uptake buffer with mannitol, as indicated. The expression of SLC22A11 was either normal (1000 ng mL^−1^ doxycycline; adjusted presentation of previous data [21]) or reduced (1.4 ng mL^−1^ doxycycline). (**C**) As in (**B**), but with 10 µM UA instead of 10 µM E3S.

**Figure 3:**
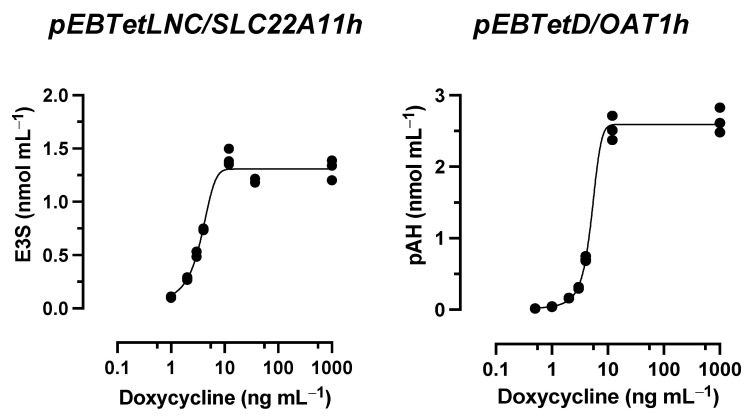
Doxycycline titration experiments. The analyte content of cell lysates was determined via LC-MS. Each symbol represents a dish. Stably transfected 293 cells expressing OAT1 or SLC22A11 (expression was induced with the indicated doxycycline concentrations) were incubated at 37 °C with 10 µM E3S (30 min) or 10 µM pAH (20 min) (*n* = 3).

During a hyperosmolarity assay verification, transport of p-aminohippuric acid (pAH), a model substrate for OAT1, was examined. Surprisingly, OAT1-expressing cells showed a slight increase in accumulation under hyperosmolar conditions after incubation with 10 µM pAH for 20 min, from 3.2 ± 0.1 nmol mg protein^−1^ to a maximum of 3.8 ± 0.1 nmol mg protein^−1^ at 400 mM mannitol (Figure 2A). This would indicate insertion into the membrane. However, because of the polar structure of pAH (Figure 1), insertion into the membrane is very unlikely. One possible reason for this discrepancy could be the particularly efficient transport of pAH by OAT1; we have previously measured, for example, 59 µL min^−1^ mg protein^−1^ (unpublished). We therefore repeated the assay with reduced expression of OAT1. The reduction was achieved by titrating doxycycline in the culture medium (Figure 3). At 3 ng mL^−1^, the accumulation of pAH by OAT1 in 293 cells was reduced to approximately 10% of the maximal accumulation at 1000 ng mL^−1^ doxycycline.

With reduced expression, pAH levels in OAT1-expressing cells decreased from 44 ± 1 pmol mg protein^−1^ to a minimum of 19 ± 1 pmol mg protein^−1^ at 800 mM mannitol (Figure 2A). This result indicates regular transport of pAH via OAT1 into the cytosol. To verify the hyperosmolarity assay in the presence of reduced expression of another transport protein, the transport of the model compounds E3S and UA via SLC22A11 was examined. After a titration experiment with E3S and several doxycycline concentrations, a doxycycline concentration of 1.4 ng mL^−1^ was determined to yield 10% uptake of E3S into cells via SLC22A11 (Figure 3). At reduced expression of SLC22A11, the UA content of cells decreased linearly with increasing mannitol concentration, whereas E3S levels increased with increasing mannitol concentration (Figure 2B,C). Thus, for SLC22A11, the results at reduced expression, in principle, conform to the established results at normal expression levels; the curves clearly differ in shape, however.

### 2.2. Comparison of IS Transport by SLC22A11 and OAT1

**Figure 4:**
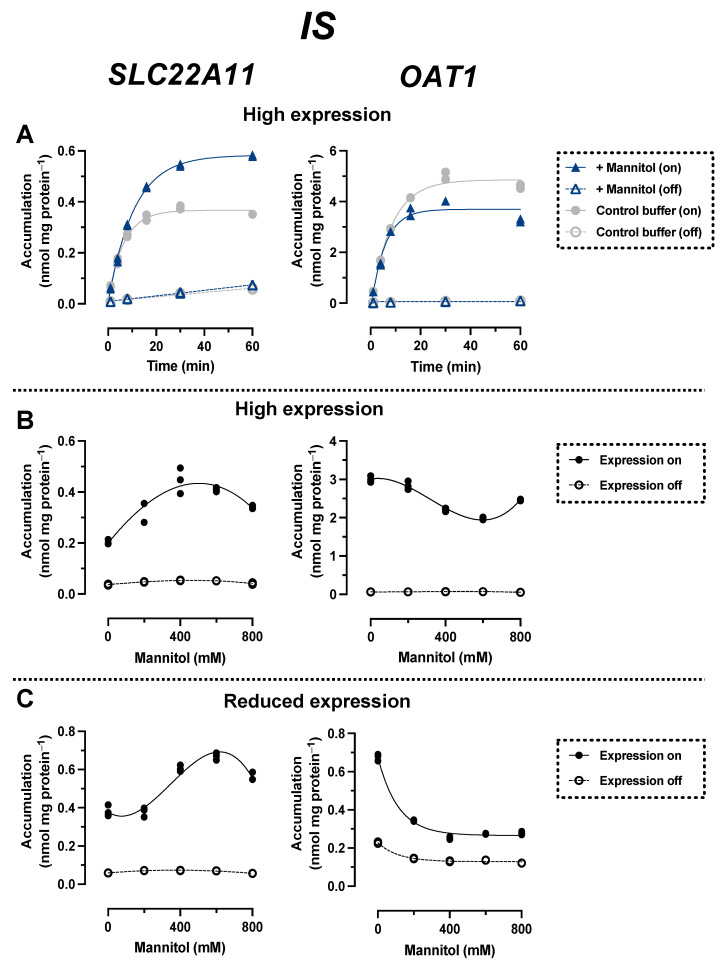
Hyperosmolarity assay reveals different mechanisms for IS transport by OAT1 and SLC22A11. The analyte content of cell lysates was determined via LC-MS. Each symbol represents a dish. (**A**) Stably transfected 293 cells with (on) or without (off) high expression (1000 ng mL^−1^ doxycycline) of OAT1 or SLC22A11 were incubated at 37 °C with 10 µM IS for the indicated time in uptake buffer (control) or in uptake buffer plus 400 mM mannitol (*n* = 2). (**B**) Stably transfected 293 cells with (on, *n* = 3) or without (off, *n* = 2) high expression (1000 ng mL^−1^ doxycycline) of OAT1 or SLC22A11 were incubated with 10 µM IS for 60 min at 37 °C in uptake buffer with mannitol, as indicated. (**C**) As in (**B**), but with reduced transporter expression (3 ng mL^−1^ doxycycline).

In time course experiments, cells expressing the transporter SLC22A11 or OAT1 (high expression) were incubated with 10 μM IS in uptake buffer with or without 400 mM mannitol (Figure 4A). The accumulation of IS by SLC22A11 over time was stimulated by hyperosmolarity; after 60 min, the accumulation of IS was 0.35 ± 0.01 nmol mg protein^−1^ (control) and 0.58 ± 0.01 nmol mg protein^−1^ (400 mM mannitol). In contrast, the accumulation of IS by OAT1 over time was inhibited by hyperosmolarity; after 60 min, the accumulation of IS was 4.6 ± 0.1 nmol mg protein^−1^ (control) and 3.3 ± 0.1 nmol mg protein^−1^ (400 mM mannitol) (Figure 4A). This implies that SLC22A11 and OAT1 differ fundamentally in their mechanism of transport of IS.

In long-term incubations (60 min) in which the uptake of IS into SLC22A11-expressing cells (high expression) was examined as a function of mannitol concentration, the IS levels increased from 0.20 ± 0.01 nmol mg protein^−1^ (0 mM mannitol) to a maximum of 0.45 ± 0.03 nmol mg protein^−1^ at about 400 mM mannitol (Figure 4B). In contrast, IS levels in OAT1-expressing cells decreased from 3.0 ± 0.1 nmol mg protein^−1^ (0 mM mannitol) to a minimum of 2.0 ± 0.1 nmol mg protein^−1^ at about 600 mM mannitol. In experiments with expression reduced to approximately 10% uptake (3 ng mL^−1^ doxycycline, see above), IS levels in OAT1-expressing cells actually decreased by more than half, from 0.68 ± 0.01 nmol mg protein^−1^ (0 mM mannitol) to 0.28 ± 0.01 nmol mg protein^−1^ (800 mM mannitol) (Figure 4C). In SLC22A11-expressing cells, again the opposite effect was observed, with an increase in IS levels from 0.38 ± 0.02 nmol mg protein^−1^ (0 mM mannitol) to a maximum of 0.67 ± 0.01 nmol mg protein^−1^ at about 600 mM mannitol (Figure 4C). These results suggest that SLC22A11 inserts IS into the plasma membrane, whereas OAT1 transports IS into the cytosol.

### 2.3. Comparison of pCS Transport by SLC22A11 and OAT1

**Figure 5:**
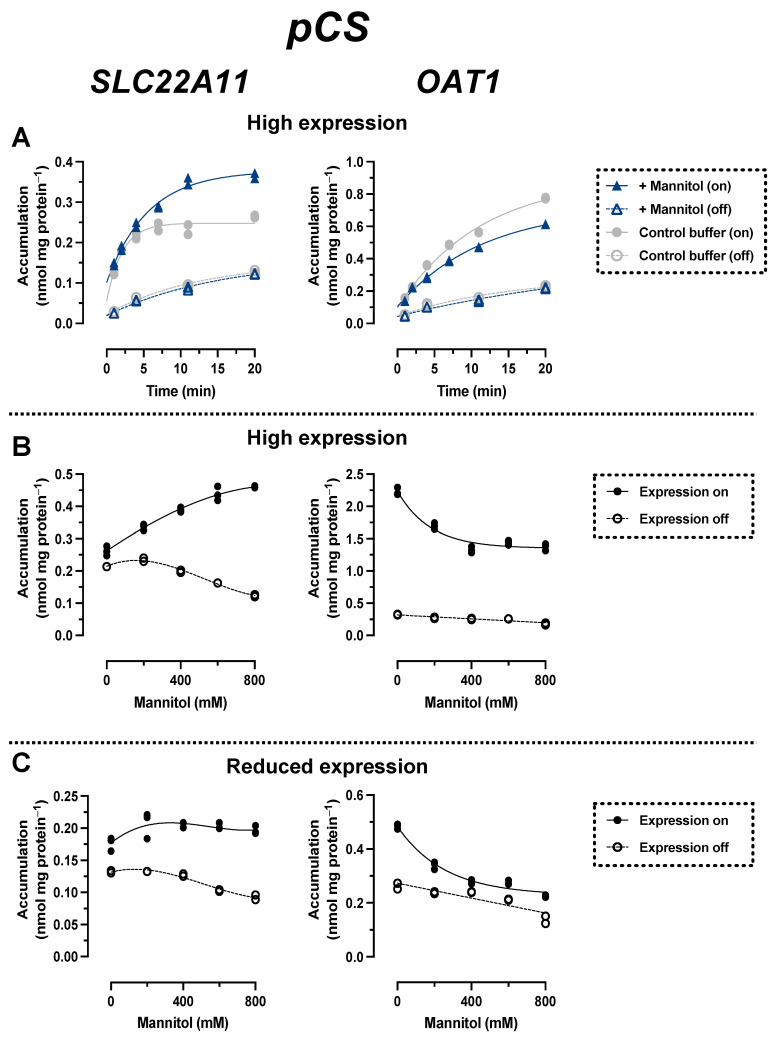
Hyperosmolarity assay reveals different mechanisms for pCS transport by OAT1 and SLC22A11. The analyte content of cell lysates was determined via LC-MS. Each symbol represents a dish. (**A**) Stably transfected 293 cells with (on) or without (off) high expression (1000 ng mL^−1^ doxycycline) of OAT1 or SLC22A11 were incubated at 37 °C with 10 µM pCS for the indicated time in uptake buffer (control) or in uptake buffer plus 400 mM mannitol (*n* = 2). (**B**) Stably transfected 293 cells with (on, *n* = 3) or without (off, *n* = 2) high expression (1000 ng mL^−1^ doxycycline) of OAT1 or SLC22A11 were incubated with 10 µM pCS for 10 min (SLC22A11) or 60 min (OAT1) at 37 °C in uptake buffer with mannitol, as indicated. (**C**) As in (**B**), but with reduced expression (3 ng mL^−1^ doxycycline).

In time course experiments (high expression), the accumulation of pCS by SLC22A11 over time was stimulated by hyperosmolarity; after 20 min, the accumulation of pCS was 0.26 ± 0.01 nmol mg protein^−1^ (control) and 0.37 ± 0.01 nmol mg protein^−1^ (400 mM mannitol) (Figure 5A). In contrast, the accumulation of pCS by OAT1 over time was inhibited by hyperosmolarity; after 20 min, the accumulation of pCS was 0.77 ± 0.01 nmol mg protein^−1^ (control) and 0.61 ± 0.01 nmol mg protein^−1^ (400 mM mannitol) (Figure 5). These results are similar to those obtained for IS (see above) and suggest that there are also different types of transport mechanism for pCS via SLC22A11 and OAT1.

In incubations in which the uptake of pCS into SLC22A11-expressing cells (high expression) was examined at equilibrium as a function of mannitol concentration, pCS concentrations increased from 0.26 ± 0.01 nmol mg protein^−1^ (0 mM mannitol) to 0.46 ± 0.01 nmol mg protein^−1^ (800 mM mannitol) (Figure 5B: cells were incubated here for 10 min instead of 60 min to reduce background noise since equilibrium was reached after 10 min; see Figure 5A). In OAT1-expressing cells, the opposite effect was observed, with a decrease in pCS levels from 2.2 ± 0.1 nmol mg protein^−1^ (0 mM mannitol) to 1.4 ± 0.1 nmol mg protein^−1^ (800 mM mannitol). In experiments with reduced transporter expression, pCS levels in OAT1-expressing cells decreased from 0.48 ± 0.01 nmol mg protein^−1^ (0 mM mannitol) to 0.22 ± 0.01 nmol mg protein^−1^ (800 mM mannitol) (Figure 5C). In contrast, pCS levels with reduced expression in SLC22A11-expressing cells increased slightly from 0.18 ± 0.01 nmol mg protein^−1^ (0 mM mannitol) to 0.20 ± 0.01 nmol mg protein^−1^ (800 mM mannitol) (Figure 4C). These results suggest that SLC22A11 also integrates pCS into the plasma membrane, whereas OAT1 transports pCS into the cytosol.

### 2.4. Transport of IP, IC, and IAA by SLC22A11

**Figure 6:**
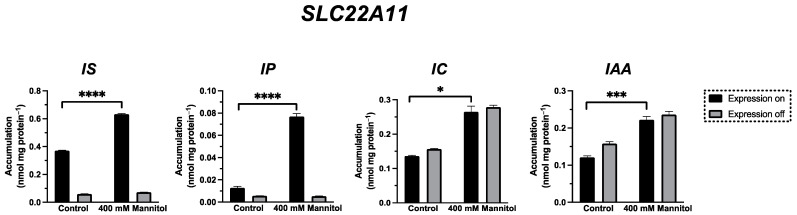
Transport of IS, IP, IC, and IAA by SLC22A11. The analyte content of cell lysates was determined via LC-MS. Stably transfected 293 cells with (on) or without (off) expression of SLC22A11 were incubated at 37 °C with 10 µM IS, 10 µM IP, 10 µM IC, or 10 µM IAA for 60 min in uptake buffer (control) or in uptake buffer plus 400 mM mannitol. Shown are the mean values with SEM (*n* = 2–3). Unpaired t-tests were performed between the control group and the 400 mM mannitol group with expression on (ns = *p* > 0.05, * = *p* ≤ 0.05; *** = *p* ≤ 0.001, **** = *p* ≤ 0.0001).

To investigate whether the sulfate residue in indoxyl sulfate (IS) can be substituted by other moieties with a negative charge in the membrane insertion by SLC22A11, the transport of the uremic toxin indole-3-acetic acid (IAA) as well as indole-3-carboxylic acid (IC) and indoxyl phosphate (IP) (Figure 1) was examined by comparing long-term incubations (60 min) of stably transfected 293 cells in uptake buffer with or without 400 mM mannitol (Figure 6). These experiments showed that the accumulation of IP in SLC22A11-expressing cells was stimulated by hyperosmolarity after 60 min; the accumulation of IP was 0.013 ± 0.001 nmol mg protein^−1^ (control) and 0.077 ± 0.003 nmol mg protein^−1^ (mannitol). This suggests that IP, like IS and pCS, is inserted into the membrane by SLC22A11. No uptake of IC or IAA into 293 cells via SLC22A11 was observed (Figure 6). 

## 3. Discussion

In this study, we examined whether the prominent uremic toxins pCS and IS are actually inserted into the plasma membrane by SLC22A11 or OAT1. This hypothesis was based on the structural similarity of pCS and IS to the steroid hormone precursor E3S (Figure 1); these compounds, when projected on a disc, consist of a large, purely hydrophobic sector and a small, very hydrophilic sector with a negative charge. Our previous data suggest that E3S is inserted into the plasma membrane by several SLC transporters, namely SLC22A11, MATE1, OAT3, SLC10A6, and SLC22A9 [18,21]. In contrast, SLC10A1 transports E3S—as a regular substrate—into the cytosol. These assumptions are based in particular on results from the hyperosmolarity assay [21], a technically simple test used to distinguish between small molecule transport into the cytosol and insertion into the plasma membrane. Here, we used this assay to compare transport of pCS and IS via SLC22A11 and OAT1.

Results for pCS and IS are consistent. Most importantly, the transport of both uremic toxins by OAT1 and SLC22A11 was fundamentally different. With SLC22A11, the uptake curves of pCS and IS (Figure 4B and Figure 5B) resemble the previous uptake curves of E3S [21]. By contrast, with OAT1, the uptake curves of pCS and IS (Figure 4B and Figure 5B) resemble the uptake curves of regular substrates, for example, the transport of UA by SLC22A11 [21]. Therefore, we suggest that SLC22A11 inserts pCS and IS into the plasma membrane, while OAT1 transports pCS and IS as regular substrates into the cytosol.

Transport to these separate compartments likely results in different mechanisms of toxicity. When pCS and IS enter the cytosol of tubule cells, for example, they can bind to and activate the human aryl hydrocarbon receptor (AHR) [6,28], inducing apoptotic and necrotic cell death [29]. In addition, oxidative stress is increased, and antioxidant capacity is decreased, leading to tubule cell damage and interstitial inflammation [29,30,31]. The damaged renal tubule activates transforming growth factor-β1 (TGF-β1) signaling, further driving interstitial inflammation and renal fibrosis [29]. Membrane insertion should have different effects. Indeed, treatment of cells with IS caused membrane damage [32] and the release of microvesicles [33].

Surprisingly, when we tested OAT1 with the model substrate pAH [34] in the hyperosmolarity assay, the uptake was *not decreased* by the addition of mannitol, but even slightly increased (Figure 2A). Clearly, because of the polar structure of pAH (Figure 1), insertion into the membrane is highly unlikely. So, what went wrong with the assay? The expression of OAT1 in our system, measured via peptide quantification, is good, but not outstanding (external Figure 7 [35]). However, the efficiency of transport of pAH by OAT1 is very high, as confirmed by our present results (Figure 2A); this is based largely on high affinity, since the K_m_ values reported at 5 and 9 μM [36,37] are rather low for a transporter. In our previous paper, we proposed that with regular substrates, the rapid decrease in accumulation could reflect a loss of accessible, active transporters from the cell surface due to the rapid reduction in the cell surface caused by hyperosmolarity [21]. However, if the transport activity (= number of active transporters × transport catalyzed by a single transporter molecule) is very high, a transport activity reserve (not detected in the isotonic control) might exist which could counteract the loss of cell surface. We tested this hypothesis by reducing the number of transporter molecules per cell in our expression system. This can be achieved easily by decreasing the concentration of the expression inducer doxycycline (Figure 3). When transporter expression was reduced to approximately 10% of maximal expression, pAH uptake indeed decreased as a function of mannitol concentration (Figure 2A), confirming regular transport of pAH by OAT1 into the cytosol. The hyperosmolarity assay at reduced transporter expression also worked to demonstrate the disparate handling of E3S and UA by SLC22A11 (Figure 2B,C); however, the curves at different transporter numbers clearly differ in shape. For pCS and IS, results are consistent at high and low expression (Figure 4C and Figure 5C). Our present results reveal that the number of transporters per cell may strongly affect the outcomes of the hyperosmolarity assay. A lower level of transporter expression would be preferred, but the activity must stand out from the background.

It is noteworthy that background accumulation of pCS (average k_in_ for transporter expression off, 0.46 µL min^−1^ mg protein^−1^) was higher compared to that of IS (0.23 µL min^−1^ mg protein^−1^) or E3S (0.24 µL min^−1^ mg protein^−1^; data from [18]). This contributes to a poorer signal-to-background ratio; in the uptake equilibrium after 10 min, accumulation of pCS mediated by SLC22A11 above the background (expression off) was much smaller (as low as f = 1.2; Figure 5B, isotonic buffer) than that observed with E3S as the substrate (f = 19; Figure 2B).

In this study, we demonstrated for the first time that pCS is a substrate of SLC22A11 (Figure 5). The efficiency of transport of pCS, extracted from the time courses of accumulation (Figure 5A, isotonic buffer; delta of k_in_ values for expression on and off), was 7.8 ± 1.4 µL min^−1^ mg protein^−1^. Thus, pCS is a relevant substrate of SLC22A11 but not as good as E3S (40 ± 6 µL min^−1^ mg protein^−1^ [18]); OAT1—which does not transport E3S [18]—translocates pCS less efficiently (3.6 ± 0.5 µL min^−1^ mg protein^−1^). The efficiency of transport of IS by SLC22A11 was similar to that of pCS at 5.6 ± 0.3 µL min^−1^ mg protein^−1^ (Figure 4A). For OAT1, however, IS is an excellent substrate at 55 ± 3 µL min^−1^ mg protein^−1^. The marked preference of OAT1 for IS may contribute to the fact that in CKD patients, the renal clearance of IS is approximately three times higher than the renal clearance of pCS [38].

Our data agree with a previous report where uptake and efflux of IS by human SLC22A11 *alias* OAT4 was demonstrated in transfected cells [13]; however, our interpretation is entirely different. It is unclear whether SLC22A11, which is located on the apical side of proximal tubule cells, has a role at all in moving cytosolic IS or pCS out of the cells after basolateral entry via OAT1 or whether its prime effect is to transfer the toxins from the luminal fluid into the cell membrane. Instead, MRP transporters could mediate the apical exit from the cytosol [1,2].

To investigate whether the sulfate residue in indoxyl sulfate (IS) can be substituted by other moieties with a negative charge in the membrane insertion by SLC22A11, the transport of the indole derivatives IAA, IC, and IP was examined using the hyperosmolarity assay. For IP, the uptake into 293 cells by SLC22A11 was stimulated by hyperosmolarity after 60 min of incubation (Figure 6), indicating the insertion of IP into the membrane by SLC22A11. This means that the sulfate group, which represents the polar part in the structures of E3S, pCS, and IS, is not essential for an insertion into the membrane by SLC22A11; a phosphate group is also accepted, albeit at a lower level of equilibrium accumulation. In contrast, a carboxylate moiety is not tolerated since IAA and IC were not transported into 293 cells by SLC22A11 (Figure 6).

Transporter-mediated membrane insertion of suitable compounds is apparently not limited to anionic solutes. Based on the cryo-EM structure of the organic cation transporter 3 (OCT3 *alias* EMT; gene symbol *SLC22A3*), a lateral access site (the V-site) was identified through which OCT3 could transport its hydrophobic substrates out of or into the plasma membrane [39]. Further cryo-EM structures of SLC transporters, particularly SLC22A11, would help to elucidate how small molecules enter the plasma membrane, providing important insights into the distribution of drugs and toxins in the human body.

In conclusion, our present results, obtained using the hyperosmolarity assay, reveal that SLC22A11 inserts the important uremic toxins pCS and IS into the plasma membrane. By contrast, OAT1 catalyzes movement into the cytosol. Transport to these separate compartments is expected to result in different mechanisms of toxicity.

## 4. Materials and Methods

### 4.1. Materials

Unlabeled compounds (if not noted otherwise, from Merck, Darmstadt, Germany; formerly Sigma-Aldrich, Munich, Germany) included 4-aminohippuric acid (08088), estrone-3-sulfate sodium salt (E0251), indole-3-acetic acid (7280.1, Carl Roth, Karlsruhe, Germany), indole-3-carboxylic acid (284734), indoxyl phosphate disodium salt (I5505), indoxyl sulfate potassium salt (I3875), potassium 4-methylphenyl sulfate (EN300-245072, Enamine, Kyiv, Ukraine), and uric acid sodium salt (U2875). All other chemicals were at least of analytical grade. Stock solutions, each at 10 mM, were prepared by dissolving estrone-3-sulfate (E3S) in methanol; indole-3-carboxylic acid (IC) in ethanol; 4-aminohippuric acid (pAH), indoxyl phosphate (IP), indoxyl sulfate (IS), and p-cresol sulfate (pCS) in water; and indole-3-acetic acid (IAA) and uric acid (UA) in water with 0.1 M sodium hydroxide.

### 4.2. Plasmids and cDNAs

The transporter cDNAs used in this study were of human origin. The SLC22A11 cDNA was expressed from pEBTetLNC [18], and OAT1 cDNA was expressed from the pEBTetD vector [40]. Both vectors are Epstein–Barr virus-derived plasmid vectors that allow doxycycline-inducible protein expression in human cell lines. Construction of pEBTetLNC/SLC22A11h [18] and pEBTetD/OAT1h [41] was described previously.

### 4.3. Cell Culture

A total of 293 cells (ATCC CRL-1573; also known as HEK-293 cells), a transformed cell line derived from human embryonic kidney, were grown as adherent culture in plastic culture flasks (Falcon 353110 and 353112, Becton Dickinson, Heidelberg, Germany) at 37 °C in a humidified 5% CO_2_ atmosphere. The growth medium was Dulbecco’s Modified Eagle Medium (Life Technologies 31885–023, Thermo Fisher Scientific, Dreieich, Germany) supplemented with 10% fetal bovine serum (FBS Analog, 2224SAMPLE, neoFroxx, Einhausen, Germany), 100 U mL^−1^ penicillin, and 0.1 mg mL^−1^ streptomycin (P4333, Sigma-Aldrich); in some experiments with SLC22A11, 10 μg mL^−1^ ciprofloxacin was used instead of penicillin/streptomycin. The medium was changed every 2–3 days, and the culture was split every 5 days. Stably transfected cell lines were generated, as reported previously [40], using Turbofect (R0531, Thermo Fisher Scientific, Dreieich, Germany). Since pEBTet-derived vectors [18,40] are propagated episomally, we used cell pools rather than single-cell clones. Cell culture medium always contained 3 μg mL^−1^ puromycin (13884, Cayman chemical, Ann Arbor, MI, USA) to maintain plasmids for up to 8 weeks in culture. Nevertheless, only cells whose transfection was not older than 5 weeks were used for the experiments.

### 4.4. Transport Assays

For measurement of solute uptake, cells were seeded in 6 cm diameter polystyrol dishes (83.3901, Sarstedt, Nümbrecht, Germany) precoated with 0.1 g l^−1^ poly-L-ornithine (P3655, Merck, Darmstadt, Germany) in 0.15 M boric acid-NaOH pH 8.4 and grown to a confluence of at least 70%. To turn protein expression on, cells were cultivated for at least 20 h with 1 μg mL^−1^ doxycycline (195044, MP Biomedicals, Eschwege, Germany) in growth medium unless otherwise indicated. Uptake buffer contained 125 mM NaCl, 25 mM HEPES-NaOH pH 7.4, 5.6 mM (+) glucose, 4.8 mM KCl, 1.2 mM KH_2_PO_4_, 1.2 mM CaCl_2_, and 1.2 mM MgSO_4_.

Incubations were performed in a water bath at 37 °C for 10 min or longer. Cells were washed at least twice with 4 mL of uptake buffer at 37 °C, and afterwards, incubations were started by adding 2 mL of 10 μM substrate in uptake buffer. After incubation, cells were washed three times with 4 mL of ice-cold uptake buffer, lysed for at least 20 min with 1 mL of methanol, and then stored at −20 °C. In some experiments, the buffer used for washing and uptake was modified as indicated with mannitol (M1902, Sigma-Aldrich). The protein content of MS samples was estimated from 3 paired dishes; here, 0.1% *v*/*v* Triton X-100 in 50 mM TRIS-HCl pH 7.4 was used as the lysis buffer. Protein was measured using the BCA (bicinchoninic acid) assay (Pierce; Thermo Fisher 23225, Life Technologies, Darmstadt, Germany) with bovine serum albumin as the standard.

### 4.5. LC-MS/MS

After centrifugation (2 min, 16,100× *g*, 20 °C) of thawed cell lysates, samples were transferred to glass vials, and then 10 μL (20 μL for pAH) of sample was analyzed via HPLC coupled to a triple-quadrupole mass spectrometer. The following system was used: an LC-20AD Prominence HPLC (Shimadzu, Duisburg, Germany) with a flow rate of 0.2 to 0.4 mL/min coupled to a 4000 Q TRAP (AB Sciex, Darmstadt, Germany) mass spectrometer. A blank or reference sample, which was methanol (HPLC gradient grade), was measured before and between sample measurements. The following HPLC methods were used. **E3S**, XBridge Shield RP18 column (3.5 μm, 3.0 × 100 mm; Waters); A: 10 mM ammonium acetate pH 8.9, B: methanol; 0.2 mL/min gradient flow: 70% B at 0 min, 70% B at 0.5 min, 20% B at 3 min, 20% B at 4 min, 70% B at 5 min, 70% B at 7 min. **IAA** and **IS**, Atlantis dC18 column (5 μm, 3.0 × 100 mm; Waters, Eschborn, Germany); A: 0.1% formic acid, B: acetonitrile with 0.1% formic acid; 0.3 mL/min gradient flow: 10% B at 0 min, 10% B at 0.25 min, 80% B at 3 min, 80% B at 5.5 min, 10% B at 7 min, 10% B at 10 min. **IC** and **IP**, Atlantis dC18 column; A: 0.1% formic acid, B: methanol with 0.1% formic acid; 0.25 mL/min gradient flow: 10% B at 0 min, 10% B at 0.25 min, 90% B at 3 min, 90% B at 6 min, 10% B at 7 min, 10% B at 10 min. **pAH**, Atlantis HILIC Silica column (5 μm, 3.0 × 50 mm; Waters) A: 10 mM ammonium acetate pH 4.0, B: methanol with 0.1% formic acid; 0.4 mL/min gradient flow: 80% B at 0 min, 80% B at 0.25 min, 20% B at 2 min, 20% B at 3 min, 80% B at 4 min, 80% B at 5 min. **pCS**, Atlantis dC18 column; A: 0.1% formic acid, B: methanol with 0.1% formic acid; 0.3 mL/min gradient flow: 10% B at 0 min, 10% B at 0.25 min, 80% B at 3 min, 80% B at 5.5 min, 10% B at 7 min, 10% B at 13 min. **UA**, iHILIC-(P) Classic column (5 μm, 2.1 × 100 mm; HILICON AB, Umeå, Sweden); A: 10 mM ammonium acetate pH 8.9, B: methanol; 0.2 mL/min gradient flow: 80% B at 0 min, 80% B at 0.25 min, 20% B at 4 min, 20% B at 5 min, 80% B at 9 min, 80% B at 10 min. We used atmospheric pressure ionization with positive or negative electrospray. For quantification (scan time 150 ms), the optimal collision energy for nitrogen-induced fragmentation in the second quadrupole was determined for each analyte. From the product ion spectra, the following fragmentations were chosen for selected reaction monitoring (SRM; *m*/*z* parent, *m*/*z* fragment, “N”/“P” for anion or cation detection with collision energy (V)): **E3S**, 349, 269, N42; **IAA**, 174, 130, N14; **IC**, 160, 116, N24; **IP**, 212, 79, N22; **IS**, 212, 80, N30; **pAH**, 195, 120, P15; **pCS**, 187, 107, N30; and **UA**, 167, 124, N22. For each analyte, the area of the intensity vs. time peak was integrated. Linear calibration curves were constructed (weighting 1/y^2^) from at least six standards, which were prepared using control cell lysates as solvent. Sample analyte content was calculated from the analyte peak area and the slope of the calibration curve.

### 4.6. Calculations and Statistics

Results are presented, if not indicated otherwise, as the arithmetic mean ± standard error of the mean (SEM) with *n* ≥ 2. For time course experiments, graphs were plotted using GraphPad Prism (version 9.2.0, GraphPad Software, San Diego, CA, USA) with Equation (1), where c_out_ represents the substrate concentration and k_in_ and k_out_ are rate constants.

y = offset + k_in_/k_out_ * c_out_ * [1 − exp(−k_out_ * x)] (1)

In osmolarity experiments, we either used the 3rd-order polynomial function of GraphPad Prism or simple linear regression. In doxycycline titration experiments, we used the sigmoidal, 4PL (four-parameter logistic) curve with Equation (2).

y = Bottom + (Top − Bottom)/(1 + 10 ^ ((Log EC_50_ − x) * HillSlope)) (2)

For Figure 6, unpaired *t*-tests were performed to determine significance; two-tailed *p*-values are shown in GraphPad style (ns = *p* > 0.05, * = *p* ≤ 0.05; ** = *p* ≤ 0.01; *** = *p* ≤ 0.001, **** = *p* ≤ 0.0001).

## Abbreviations

pAH, p-aminohippuric acid; CKD, chronic kidney disease; pCS, p-cresol sulfate; E3S, estrone-3-sulfate; IS, indoxyl sulfate; LC, liquid chromatography; MS, mass spectrometry; OAT1, organic anion transporter 1; UA, uric acid.

## References

[1] Clark W.R., Dehghani N.L., Narsimhan V., Ronco C. (2019). Uremic Toxins and their Relation to Dialysis Efficacy. Blood Purif..

[2] Lim Y.J., Sidor N.A., Tonial N.C., Che A., Urquhart B.L. (2021). Uremic Toxins in the Progression of Chronic Kidney Disease and Cardiovascular Disease: Mechanisms and Therapeutic Targets. Toxins.

[3] Stevens P.E., Levin A. (2013). Kidney Disease: Improving Global Outcomes Chronic Kidney Disease Guideline Development Work Group. Evaluation and Management of Chronic Kidney Disease: Synopsis of the Kidney Disease: Improving Global Outcomes 2012 Clinical Practice Guideline. Ann. Intern. Med..

[4] Chen T.K., Knicely D.H., Grams M.E. (2019). Chronic Kidney Disease Diagnosis and Management: A Review. J. Am. Med. Assoc..

[5] Itoh Y., Ezawa A., Kikuchi K., Tsuruta Y., Niwa T. (2012). Protein-bound uremic toxins in hemodialysis patients measured by liquid chromatography/tandem mass spectrometry and their effects on endothelial ROS production. Anal. Bioanal. Chem..

[6] Liu W.-C., Tomino Y., Lu K.-C. (2018). Impacts of Indoxyl Sulfate and p-Cresol Sulfate on Chronic Kidney Disease and Mitigating Effects of AST-120. Toxins.

[7] Gryp T., Vanholder R., Vaneechoutte M., Glorieux G. (2017). p-Cresyl sulfate. Toxins.

[8] Gamage N., Barnett A., Hempel N., Duggleby R.G., Windmill K.F., Martin J.L., McManus M.E. (2005). Human Sulfotransferases and Their Role in Chemical Metabolism. Toxicol. Sci..

[9] Leong S.C., Sirich T.L. (2016). Indoxyl Sulfate—Review of Toxicity and Therapeutic Strategies. Toxins.

[10] Banoglu E., Jha G.G., King R.S. (2001). Hepatic microsomal metabolism of indole to indoxyl, a precursor of indoxyl sulfate. Eur. J. Drug Metab. Pharmacokinet..

[11] Banoglu E., King R.S. (2002). Sulfation of indoxyl by human and rat aryl (phenol) sulfotransferases to form indoxyl sulfate. Eur. J. Drug Metab. Pharmacokinet..

[12] Enomoto A., Takeda M., Tojo A., Sekine T., Cha S.H., Khamdang S., Takayama F., Aoyama I., Nakamura S., Endou H. (2002). Role of Organic Anion Transporters in the Tubular Transport of Indoxyl Sulfate and the Induction of its Nephrotoxicity. J. Am. Soc. Nephrol..

[13] Enomoto A., Takeda M., Taki K., Takayama F., Noshiro R., Niwa T., Endou H. (2003). Interactions of human organic anion as well as cation transporters with indoxyl sulfate. Eur. J. Pharmacol..

[14] Miyamoto Y., Watanabe H., Noguchi T., Kotani S., Nakajima M., Kadowaki D., Otagiri M., Maruyama T. (2011). Organic anion transporters play an important role in the uptake of p-cresyl sulfate, a uremic toxin, in the kidney. Nephrol. Dial. Transplant..

[15] Nigam S.K., Bush K.T., Martovetsky G., Ahn S.-Y., Liu H.C., Richard E., Bhatnagar V., Wu W. (2015). The Organic Anion Transporter (OAT) Family: A Systems Biology Perspective. Physiol. Rev..

[16] Cha S.H., Sekine T., Kusuhara H., Yu E., Kim J.Y., Kim D.K., Sugiyama Y., Kanai Y., Endou H. (2000). Molecular Cloning and Characterization of Multispecific Organic Anion Transporter 4 Expressed in the Placenta. J. Biol. Chem..

[17] Hagos Y., Stein D., Ugele B., Burckhardt G., Bahn A. (2007). Human Renal Organic Anion Transporter 4 Operates as an Asymmetric Urate Transporter. J. Am. Soc. Nephrol..

[18] Skwara P., Schömig E., Gründemann D. (2017). A novel mode of operation of SLC22A11: Membrane insertion of estrone sulfate versus translocation of uric acid and glutamate. Biochem. Pharmacol..

[19] Hearn E.M., Patel D.R., Lepore B.W., Indic M., Van den Berg B. (2009). Transmembrane passage of hydrophobic compounds through a protein channel wall. Nature.

[20] Agboh K., Lau C.H.F., Khoo Y.S.K., Singh H., Raturi S., Nair A.V., Howard J., Chiapello M., Feret R., Deery M.J. (2018). Powering the ABC multidrug exporter LmrA: How nucleotides embrace the ion-motive force. Sci. Adv..

[21] Müller J.P., Keufgens L., Gründemann D. (2021). Hyperosmolarity stimulates transporter-mediated insertion of estrone sulfate into the plasma membrane, but inhibits the uptake by SLC10A1 (NTCP). Biochem. Pharmacol..

[22] Loh J., Chuang M.-C., Lin S.-S., Joseph J., Su Y.-A., Hsieh T.-L., Chang Y.-C., Liu A.P., Liu Y.-W. (2019). Acute decrease in plasma membrane tension induces macropinocytosis *via* PLD2 activation. J. Cell Sci..

[23] Kang Y.-S., Ko Y.-G., Seo J.-S. (2000). Caveolin Internalization by Heat Shock or Hyperosmotic Shock. Exp. Cell Res..

[24] Dupont S., Beney L., Ritt J.-F., Lherminier J., Gervais P. (2010). Lateral reorganization of plasma membrane is involved in the yeast resistance to severe dehydration. Biochim. Biophys. Acta (BBA)-Biomembr..

[25] Wang S., Singh R.D., Godin L., Pagano R.E., Hubmayr R.D., Cong X., Li C., Zhao X., Nagre N., Kellett T. (2011). Endocytic response of type I alveolar epithelial cells to hypertonic stress. Am. J. Physiol. Cell. Mol. Physiol..

[26] Sommerfeld A., Mayer P.G., Cantore M., Häussinger D. (2015). Regulation of plasma membrane localization of the Na+-taurocholate cotransporting polypeptide (Ntcp) by hyperosmolarity and tauroursodeoxycholate. J. Biol. Chem..

[27] Le Roux A.-L., Quiroga X., Walani N., Arroyo M., Roca-Cusachs P. (2019). The plasma membrane as a mechanochemical transducer. Philos. Trans. R. Soc. B Biol. Sci..

[28] Schroeder J.C., DiNatale B.C., Murray I.A., Flaveny C.A., Liu Q., Laurenzana E.M., Lin J.M., Strom S.C., Omiecinski C.J., Amin S. (2009). The Uremic Toxin 3-Indoxyl Sulfate Is a Potent Endogenous Agonist for the Human Aryl Hydrocarbon Receptor. Biochemistry.

[29] Cheng T.-H., Ma M.-C., Liao M.-T., Zheng C.-M., Lu K.-C., Liao C.-H., Hou Y.-C., Liu W.-C., Lu C.-L. (2020). Indoxyl Sulfate, a Tubular Toxin, Contributes to the Development of Chronic Kidney Disease. Toxins.

[30] Motojima M., Hosokawa A., Yamato H., Muraki T., Yoshioka T. (2003). Uremic toxins of organic anions up-regulate PAI-1 expression by induction of NF-kappa B and free radical in proximal tubular cells. Kidney Int..

[31] Edamatsu T., Fujieda A., Itoh Y. (2018). Phenyl sulfate, indoxyl sulfate and p-cresyl sulfate decrease glutathione level to render cells vulnerable to oxidative stress in renal tubular cells. PLoS ONE.

[32] Idziak M., Pędzisz P., Burdzińska A., Gala K., Pączek L. (2014). Uremic toxins impair human bone marrow-derived mesenchymal stem cells functionality in vitro. Exp. Toxicol. Pathol..

[33] Alique M., Bodega G., Corchete E., García-Menéndez E., de Sequera P., Luque R., Rodríguez-Padrón D., Marqués M., Portolés J., Carracedo J. (2020). Microvesicles from indoxyl sulfate-treated endothelial cells induce vascular calcification in vitro. Comput. Struct. Biotechnol. J..

[34] Burckhardt G., Wolff N.A., Sweet D.H., Chan L.M.S., Walden R., Yang X.-P., Miller D.S., Pritchard J.B., Grassl S.M., Zhang X. (2000). Structure of renal organic anion and cation transporters. Am. J. Physiol. Physiol..

[35] Tschirka J., Bach M., Kisis I., Lemmen J., Gnoth M.J., Gründemann D. (2020). Transporter tandems: Precise tools for normalizing active transporter in the plasma membrane. Biochem. J..

[36] Hosoyamada M., Sekine T., Kanai Y., Endou H., Breljak D., Ljubojević M., Hagos Y., Micek V., Eror D.B., Madunić I.V. (1999). Molecular cloning and functional expression of a multispecific organic anion transporter from human kidney. Am. J. Physiol. Physiol..

[37] Lu R., Chan B.S., Schuster V.L., Henjakovic M., Hagos Y., Krick W., Burckhardt G., Burckhardt B.C., Hu Q.-H., Wang C. (1999). Cloning of the human kidney PAH transporter: Narrow substrate specificity and regulation by protein kinase C. Am. J. Physiol. Physiol..

[38] Poesen R., Viaene L., Verbeke K., Claes K., Bammens B., Sprangers B., Naesens M., Vanrenterghem Y., Kuypers D., Evenepoel P. (2013). Renal Clearance and Intestinal Generation of p-Cresyl Sulfate and Indoxyl Sulfate in CKD. Clin. J. Am. Soc. Nephrol..

[39] Khanppnavar B., Maier J., Herborg F., Gradisch R., Lazzarin E., Luethi D., Yang J.-W., Qi C., Holy M., Jäntsch K. (2022). Structural basis of organic cation transporter-3 inhibition. Nat. Commun..

[40] Bach M., Grigat S., Pawlik B., Fork C., Utermöhlen O., Pal S., Banczyk D., Lazar A., Schömig E., Gründemann D. (2007). Fast set-up of doxycycline-inducible protein expression in human cell lines with a single plasmid based on Epstein-Barr virus replication and the simple tetracycline repressor. FEBS J..

[41] Fork C., Bauer T., Golz S., Geerts A., Weiland J., Del Turco D., Schömig E., Gründemann D. (2011). OAT2 catalyses efflux of glutamate and uptake of orotic acid. Biochem. J..

